# Supplementary material for: Cortactin Contributes to Activity-Dependent Modulation of Spine Actin Dynamics and Spatial Memory Formation
Source: Cells. 2021 Jul 20;10(7):1835. doi: 10.3390/cells10071835 (PMC8303107; doi:10.3390/cells10071835)
Supplement: Supplementary file 1 [file cells-10-01835-s001.zip › cells-1282109-supplementary.pdf]

Supplementary Information

Table S1 – Statistical Information.

**Figure**	**Treatment & Statistics**				
**Fig.1A**	Normal distribution?	Kolmogorov-Smirnov Test		Passed: No	
		Kruskal-Wallis test with post-Hoc Dunn's test			
		**Wildtype**	***Cttn* KO**		
	Border	86.436 ± 1.871 %	86.065 ± 2.277 %		
		WT middle p<0.05	KO middle p<0.05		
		WT center p<0.001	KO center p<0.001		
	Middle	10.561 ± 1.292 %	10.356 ± 1.569 %		
	Center	2.980 ± 0.746 %	3.577 ± 0.788 %		

**Fig.1B**	Normal distribution?	Kolmogorov-Smirnov Test		Passed: Yes	
	Two-Way ANOVA	Interaction	F(4.12,61.2) = 8.66	p = 0.593	df = 4.12
	Repeated Measures	Genotype	F(1,20) = 3.14	p = 0.092	df = 1
		Training Time	F(7,104) = 0.796	p < 0.001	df = 7
	**Escape Latency**	**Wildtype**	***Cttn* KO**		
	Training Day 1	27.658 ± 5.104 s	38.681 ± 4.448 s		
	Training Day 2	20.544 ± 2.830 s	26.472 ± 4.464 s		
	Training Day 3	17.842 ± 2.715 s	24.940 ± 6.263 s		
	Training Day 4	12.058 ± 3.874 s	17.545 ± 3.287 s		
	Training Day 5	15.575 ± 5.625 s	28.980 ± 2.719 s		
	Training Day 6	12.792 ± 4.834 s	19.935 ± 3.198 s		
	Training Day 7	12.350 ± 4.846 s	16.590 ± 4.577 s		
	Training Day 8	7.575 ± 1.816 s	13.730 ± 2.761 s		

**Fig.1C**	Normal distribution?	Kolmogorov-Smirnov Test		Passed: No
	**Platform crossings**	**Wildtype**	***Cttn* KO**	
	Probe Trial Day 3	2.333 ± 0.408	1.375 ± 0.324	
	Probe Trial Day 6	2.667 ± 0.421	1.400 ± 0.400	
	Probe Trial Day 9	3.000 ± 0.632	2.400 ± 0.600	

**Fig.1D**	Normal distribution?	Kolmogorov-Smirnov Test		Passed: Yes	
	Two-Way ANOVA	Interaction	F(1,64) = 8.047	p = 0.006	df=1
	Sidak's Multiple Comparisons	Genotype	F(1,64) = 1.989	p = 0.163	df=1
		Quadrants	F(1,64) = 15.54	p < 0.001	df=1
	**Quadrant Preference**	**Wildtype**	***Cttn* KO**		
	Target Quadrant	39.333 ± 3.660 %	27.365 ± 3.520 %		
		WT OQ p<0.01			
	Other Quadrants	20.520 ± 1.945 %	23.122 ± 2.360 %		

Figure	Treatment & Statiscs				
**Fig.1E**	Normal distribution?	Kolmogorov-Smirnov Test		Passed: Yes	
	Two-Way ANOVA	Interaction	F(1,40) = 4.650	p = 0.037	df=1
	Sidak's Multiple Comparisons	Genotype	F(1,40) = 1.176	p = 0.285	df=1
		Quadrants	F(1,40) = 21.97	p < 0.001	df=1
	**Quadrant Preference**	**Wildtype**	***Cttn* KO**		
	Target Quadrant	43.148 ± 3.891 %	31.690 ± 5.513 %		
		WT OQ p<0.001			
	Other Quadrants	18.951 ± 1.792 %	22.741 ± 3.122 %		
**Fig.1F**	Normal distribution?	Kolmogorov-Smirnov Test		Passed: Yes	
	Two-Way ANOVA	Interaction	F(1,40) = 0.918	p = 0.344	df=1
	Sidak's Multiple Comparisons	Genotype	F(1,40) = 0.225	p = 0.638	df=1
		Quadrants	F(1,40) = 61.77	p < 0.001	df=1
	**Quadrant Preference**	**Wildtype**	***Cttn* KO**		
	Target Quadrant	49.148 ± 3.869 %	43.910 ± 5.599 %		
		WT OQ p<0.001	KO OQ p<0.001		
	Other Quadrants	16.913 ± 2.013 %	18.681 ± 3.869 %		
**Fig.2A**	Normal distribution?	Kolmogorov-Smirnov Test			Passed: Yes
	**Stimulus Intensity**	**Wildtype**	***Cttn* KO**		
	25 µA	-0.521 ± 0.062 mV/ms	-0.516 ± 0.074 mV/ms		
	50 µA	-0.977 ± 0.147 mV/ms	-0.883 ± 0.125 mV/ms		
	75 µA	-1.484 ± 0.288 mV/ms	-1.146 ± 0.143 mV/ms		
	100 µA	-1.870 ± 0.362 mV/ms	-1.488 ± 0.197 mV/ms		
	125 µA	-2.201 ± 0.398 mV/ms	-1.773 ± 0.241 mV/ms		
	150 µA	-2.440 ± 0.421 mV/ms	-1.998 ± 0.269 mV/ms		
	175 µA	-2.675 ± 0.442 mV/ms	-2.205 ± 0.294 mV/ms		
	200 µA	-2.804 ± 0.462 mV/ms	-2.300 ± 0.296 mV/ms		
	225 µA	-2.959 ± 0.281 mV/ms	-2.410 ± 0.323 mV/ms		
	250µA	-3.022 ± 0.474 mV/ms	-2.480 ± 0.307 mV/ms		
**Fig.2B**	Normal distribution?	Kolmogorov-Smirnov Test			Passed: Yes
	**Fiber Volley Amplitude**	**Wildtype**	***Cttn* KO**		
	0.1 mV	-0.137 ± 0.010 mV/ms	-0.129 ± 0.011 mV/ms		
	0.2 mV	-0.168 ± 0.013 mV/ms	-0.163 ± 0.021 mV/ms		
	0.3 mV	-0.238 ± 0.020 mV/ms	-0.219 ± 0.019 mV/ms		
	0.4 mV	-0.270 ± 0.021 mV/ms	-0.215 ± 0.020 mV/ms		
	0.5 mV	-0.315 ± 0.034 mV/ms	-0.254 ± 0.023 mV/ms		
	0.6 mV	-0.333 ± 0.033 mV/ms	-0.268 ± 0.024 mV/ms		
	0.7 mV	-0.379 ± 0.049 mV/ms	-0.313 ± 0.022 mV/ms		
	0.8 mV	-0.443 ± 0.048 mV/ms	-0.358 ± 0.023 mV/ms		

Figure	Treatment & Statiscs			
**Fig.2C**	Normal distribution?	Kolmogorov-Smirnov Test		Passed: Yes
	**ISI**	**Wildtype**	***Cttn* KO**	
	160 ms	131.736 ± 6.900 %	137.916 ± 4.670 %	
	80 ms	171.860 ± 4.306 %	166.184 ± 7.126 %	
	40 ms	175.633 ± 10.041 %	187.450 ± 12.023 %	
	20 ms	251.070 ± 33.038 %	261.364 ± 28.971 %	
	10 ms	286.319 ± 41.884 %	275.367 ± 30.732 %	
**Fig.2D**	Normal distribution?	Kolmogorov-Smirnov Test		Passed: Yes
		**Wildtype**	***Cttn* KO**	
	Last 5′ of LTP	134.753 ± 0.333 %<br>*Cttn* KO $p < 0.001$<br>Student's t-Test	125.037 ± 0.795 %	
**Fig.3B**	Normal distribution?	Kolmogorov-Smirnov Test		Passed: No
	Kruskal-Wallis test with post-Hoc Dunn's test			
	**Turnover Time**	**Wildtype**	***Cttn* KO**	
	DIV14	59.770 ± 6.422 s	51.549 ± 2.880 s	
	DIV21	40.636 ± 2.943 s	36.698 ± 1.990 s<br>KO DIV14 $p<0.01$	
	**Dynamic Fraction**	**Wildtype**	***Cttn* KO**	
	DIV14	0.819 ± 0.028	0.827 ± 0.028	
	DIV21	0.799 ± 0.018	0.820 ± 0.019	
**Fig.3D**	Normal distribution?	Kolmogorov-Smirnov Test		Passed: Yes
	**abs. µm change**	**Wildtype**	***Cttn* KO**	
	DIV14	0.147 ± 0.010 µm	0.172 ± 0.009 µm	
	DIV21	0.146 ± 0.010 µm	0.161 ± 0.011 µm	
	**mean µm change**	**Wildtype**	***Cttn* KO**	
	DIV14	0.006 ± 0.008 µm	0.007 ± 0.005 µm	
	DIV21	-0.003 ± 0.007 µm	-0.003 ± 0.003 µm	
**Fig.3E**	Normal distribution?	Kolmorogov-Smirnov Test		Passed: No
	**abs. length change**	**Wildtype**	***Cttn* KO**	
	DIV14	0.220 ± 0.037 µm	0.195 ± 0.015 µm	
	DIV21	0.166 ± 0.007 µm	0.178 ± 0.016 µm	
	**mean length change**	**Wildtype**	***Cttn* KO**	
	DIV14	-0.020 ± 0.011 µm	-0.012 ± 0.005 µm	
	DIV21	0.016 ± 0.006 µm	0.007 ± 0.005 µm	

Figure	Treatment & Statiscs				
**Fig.4A**	Normal distribution?	Kolmogorov-Smirnov Test		Passed: Yes	
	**Spine density**	**Wildtype**	***Cttn* KO**		
	Spines/µM	1.122 ± 0.049	1.195 ± 0.086		
**Fig.4B**	Normal distribution?	Kolmogorov-Smirnov Test		Passed: Yes	
	Two-Way ANOVA	Interaction	F(1,56) = 22.54	p < 0.001	df=1
	Sidak's Multiple Comparisons	Genotype	F(1,56) = 10.66	p = 0.002	df=1
		Treatment	F(1,56) = 8.225	p = 0.006	df=1
	**Spine head diameter**	**Wildtype**	***Cttn* KO**		
	Ctrl	0.553 ± 0.011 µm	0.583 ± 0.008 µm		
	60′ post cLTP	0.685 ± 0.013 µm<br>WT Ctrl p < 0.001	0.599 ± 0.013 µm		
**Fig.4E**	Normal distribution?	Kolmogorov-Smirnov Test		Passed: No	
		Kruskal-Wallis test with post-Hoc Dunn's test			
	**Turnover Time**	**Wildtype**	***Cttn* KO**		
	Ctrl	51.759 ± 2.828 s	46.671 ± 3.069 s		
	15′ post cLTP	65.612 ± 5.557 s	75.897 ± 8.123 s<br>KO Ctrl p<0.01		
	**Dynamic Fraction**	**Wildtype**	***Cttn* KO**		
	Ctrl	0.787 ± 0.015	0.763 ± 0.012		
	15′ post cLTP	0.699 ± 0.023<br>WT Ctrl p<0.05	0.835 ± 0.058		
